# A rare case report of idiopathic renal infarction initially misdiagnosed as acute gastroenteritis

**DOI:** 10.1097/MD.0000000000043081

**Published:** 2025-07-04

**Authors:** Libin Liu, Jingjiao Chen, Xiaomeng Wang, Na Shi, Yi Hu

**Affiliations:** a Department of Gastroenterology, Affiliated Hospital of Qingdao University, Qingdao, Shandong Province, China; b Department of Health Care/Geriatrics, Affiliated Hospital of Qingdao University, Qingdao, Shandong Province, China.

**Keywords:** case report, gastroenteritis, idiopathic, renal infarction

## Abstract

**Rationale:**

Idiopathic renal infarction refers to the sudden necrosis of kidney tissue due to a loss of blood supply, without an identifiable or specific underlying cause. It is a rare and often misdiagnosed condition owing to its nonspecific symptoms, with clinical manifestations similar to gastrointestinal or urinary disorders.

**Patient concerns:**

A 40-year-old male with no significant past medical history presented with sudden-onset right lower abdominal pain, nausea, vomiting, and diarrhea.

**Diagnoses:**

Initial laboratory and imaging studies, including abdominal computed tomography (CT), did not reveal any specific abnormalities, leading to a diagnosis of acute gastroenteritis. However, after a second episode of similar symptoms, further investigations, including contrast-enhanced CT, revealed evidence of renal infarction.

**Interventions:**

The patient was treated with anticoagulation therapy and infection control, and his symptoms improved gradually.

**Outcomes:**

After 6 months of follow-up, the patient had not recurred.

**Lessons:**

Although the condition is rare, idiopathic renal infarction can occur in otherwise healthy individuals, presenting with symptoms commonly associated with gastrointestinal disorders. Contrast-enhanced CT is more reliable for the diagnosis of renal infarction. Early aggressive anticoagulation is fundamental to treating idiopathic renal infarction.

## 1. Introduction

Acute renal infarction is a rare condition, with previous reports indicating an incidence rate of approximately 0.004% to 0.007%.^[1,2]^ The primary cause of acute renal infarction is cardiac disease, with atrial fibrillation identified as the most common cause (accounting for up to 65%), followed by valvular heart disease and cardiomyopathy.^[1,3–5]^ Other contributing factors include atherosclerosis, arterial dissection, antiphospholipid syndrome, connective tissue diseases, vasculitis, trauma, severe inflammatory response syndrome, and hypercoagulable states, all of which may lead to renal artery thrombosis.^[6–8]^ Hyperhomocysteinemia may also contribute to the pathogenesis of renal artery thrombosis.^[9]^ In this report, we present an extremely rare case of acute renal infarction without any underlying health conditions. The patient initially presented with symptoms of gastrointestinal disorders.

## 2. Case presentation

**Figure 1.**
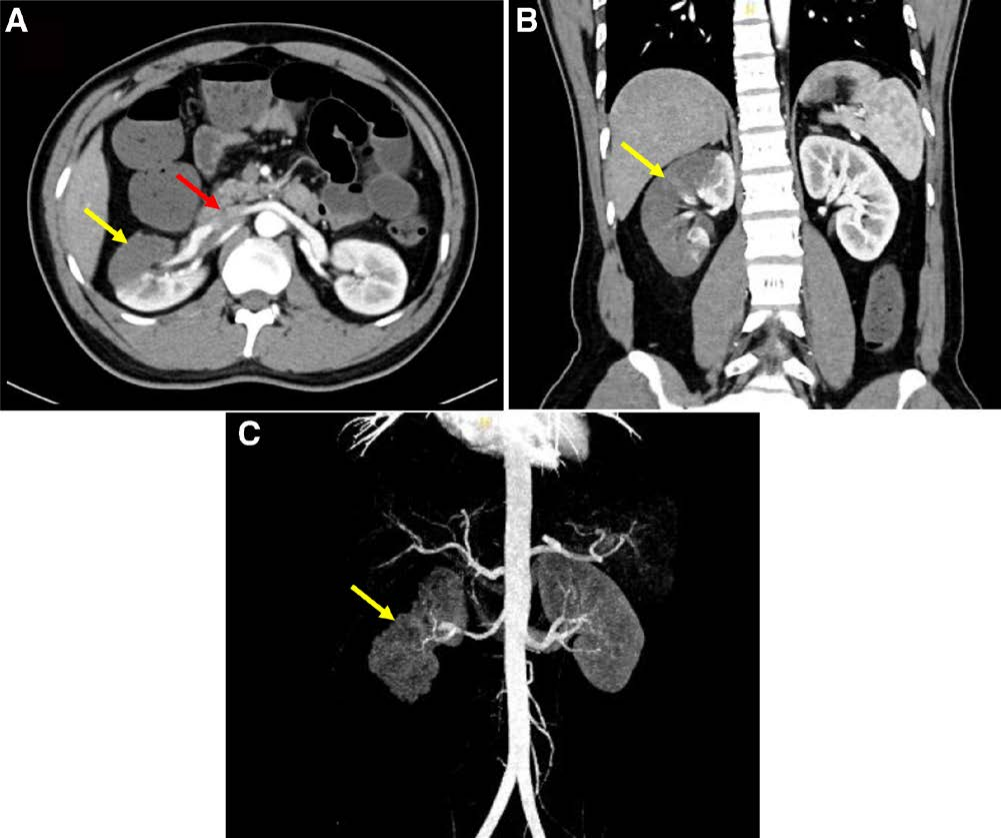
Contrast-enhanced CT image of the patient showing a right renal infarction. (A) Axial section; (B) sagittal section; (C) vascular reconstruction. A patchy low-density area in the right kidney (yellow arrow). A strip-like low-density area was noted in the right renal artery region (red arrow).

A 40-year-old male presented with sudden-onset right lower abdominal pain, bloating, sweating, nausea, vomiting, and diarrhea (5 times with loose stools). He also had a fever of 38.4 °C but no hematochezia, melena, or chest tightness. Physical examination revealed right lower abdominal tenderness and rebound tenderness. Laboratory tests showed that white blood cell count (WBC) 15.70 × 10⁹/L, neutrophil percentage 94.5%, C-reactive protein (CRP) 0.05 mg/L, creatinine 102.5 μmol/L (normal range: 57–97 μmol/L), serum aspartate aminotransferase 133 U/L (normal range: 14–50 U/L), serum alanine aminotransferase 98 U/L (normal range: 9–50 U/L), and glucose in urine 3+. Abdominal CT and ultrasonography revealed no abnormalities. A diagnosis of acute gastroenteritis was considered. The patient was treated with ceftriaxone for 3 days. His abdominal pain was alleviated, fever remitted, and WBC decreased, after which the patient was discharged and continued oral cefdinir therapy. Three days later, the patient returned with similar symptoms, including right lower abdominal pain radiating to the periumbilical region, bloating, nausea, and vomiting. Physical examination revealed tenderness in the lower right abdomen and around the umbilicus. Laboratory tests showed that WBC 10.98 × 10⁹/L, neutrophil percentage 72.4%, CRP 59.07 mg/L, creatinine 127.1 μmol/L. A repeat abdominal CT scan revealed no significant abnormalities. The patient was treated with ceftriaxone for infection and pain management with raceanisodamine, phloroglucinol, and pethidine hydrochloride. However, the pain persisted, and the patient experienced cessation of bowel movements and flatus. Contrast-enhanced abdominal CT was subsequently performed, revealing a patchy low-density area in the right kidney, suggestive of renal infarction. A strip-like low-density area was noted in the right renal artery region, raising suspicion of partial renal artery embolism, and intestinal obstruction was considered (Fig. 1). The final diagnosis was renal infarction. The patient was started on anticoagulation therapy (low molecular weight heparin 5000 IU every 12 hours) was initiated, along with combined infection control (ceftriaxone), acid suppression (pantoprazole), blood flow improvement (papaverine, prostaglandin E1), liver protection (polyunsaturated phosphatidylcholine, magnesium isoglycyrrhizinate) and intravenous nutrition.

Efforts were made to identify the cause of the infarction. The patient had no history of hypertension, heart disease, diabetes, or cerebrovascular disease, and was physically healthy. He had been smoking for 10 years, averaging 5 cigarettes per day, and occasionally drank alcohol. Family history included hyperlipidemia in the father and hypertension and diabetes in the mother. The following tests were performed: urine occult blood 1+, urine protein 1+; red blood cells in urine 14.00 cells/μL, lactate dehydrogenase (LDH) 555 U/L (120–250 U/L), homocysteine 16.42 μmol/L (0–15 μmol/L). Tests for antiphospholipid antibodies, blood lipid analysis, blood glucose, troponin I/T, rheumatoid factor, antineutrophil cytoplasmic antibodies, extractable nuclear antigens antibody profile, and antinuclear antibodies were all within normal limits. No significant abnormalities were observed on the cardiac ultrasound. Right heart ultrasound contrast imaging revealed no contrast microbubbles in the left heart (negative), excluding structural heart abnormalities. Ultrasound of lower extremity vessels, cervical vessels, brain MRI, magnetic resonance angiography of cerebral arteries, abdominal aortic color doppler ultrasound, and electrocardiogram showed no significant abnormalities. After 1 week of treatment, doppler ultrasound of both kidneys showed that the renal artery blood flow bundle width at the right renal hilum was about 0.6 cm, and at the left renal hilum was about 0.9 cm. The patient’s symptoms gradually improved. Upon discharge, the patient continued oral rivaroxaban (10 mg daily). One month later, follow-up tests showed normal blood routine, and no abnormalities in WBC, procalcitonin, and liver and kidney function. Doppler ultrasound of both kidneys showed no significant changes in the renal artery blood flow bundle compared with prior results. After 6 months of follow-up, the patient did not experience recurrence.

## 3. Discussion

In this case, the patient had no history of cardiac disease, normal blood lipid levels, and no abnormalities observed on ultrasound examinations of the heart and peripheral arteries. Immunological markers, including antinuclear antibodies and rheumatoid factor, were also normal. Vascular reconstruction of CT revealed occlusion of a single renal branch, with no significant abnormalities in the other vessels. Aside from a history of smoking, the etiology of the patient was negative, leading to a diagnosis of idiopathic renal infarction, a very rare condition.

The clinical manifestations of acute renal infarction are nonspecific, with persistent flank and abdominal pain being the most common symptom, followed by nausea, vomiting, and fever.^[4,10,11]^ These symptoms can be easily confused with those of ureteral stones, pyelonephritis, appendicitis, gastroenteritis, biliary stones, and pelvic masses with torsion. On physical examination, costovertebral angle tenderness is a characteristic finding in patients with acute renal infarction. However, individuals with small vessel thrombosis may present with minimal symptoms. Renal infarction may also induce acute hypertension, although this elevation in blood pressure is often transient. Due to the rarity and nonspecific nature of its symptoms, diagnosis is often delayed or misdiagnosed.

Laboratory tests for acute renal infarction lack specificity and sensitivity. Common laboratory abnormalities include elevated WBC, CRP, abnormal renal and liver function tests, hematuria (both gross and microscopic), proteinuria, and elevated lactate LDH levels. Notably, elevated LDH is highly sensitive for detecting acute renal infarction, with 93% to 100% of patients showing increased serum LDH levels.^[1–4,12]^ Although elevated LDH levels are nonspecific at the site of tissue necrosis, such an increase should not be observed in patients with urinary stones. Therefore, in cases with persistent flank and abdominal pain but no evidence of urolithiasis, serum LDH levels should be assessed. In this case, LDH was not measured early in the course of the disease, but upon confirming renal infarction, we found that the patient’s serum LDH levels were significantly elevated. Studies have shown that serum creatinine level is a reliable indicator of the severity of renal infarction and length of hospitalization. Elevated serum creatinine levels correlate with more extensive infarction and greater renal damage.^[12]^ In this case, serum creatinine was elevated early in the course of the disease, but normalized 1 week after treatment, suggesting a favorable prognosis for the patient.

Ultrasound examination can assess the kidney’s morphology, size, and structure, detect abnormal echo regions within the kidneys, and exclude conditions such as urolithiasis or urinary obstruction. Color doppler ultrasound can evaluate renal blood flow and detect thrombosis or stenosis in the renal arteries and veins. In renal infarction patients, color doppler ultrasound typically shows reduced or absent blood flow in the infarcted region. Ultrasound is noninvasive, convenient, and quick, but its sensitivity is lower for early renal infarction or small infarctions and can be significantly influenced by the operator’s experience and technical skill. Non-contrast CT is the first-choice diagnostic tool for renal colic, as it can detect almost all urinary stones. However, it is not effective in identifying thromboembolic diseases, and renal infarction may be missed. Contrast-enhanced CT is more reliable for diagnosing renal infarction, as it reveals wedge-shaped or circular low-density infarcts, that do not enhance post-contrast.^[13]^ Additionally, renal CT angiography can assess the renal blood supply and detect stenosis, thrombosis, or other vascular abnormalities.^[14]^ Renal arteriography is considered the “gold standard” for diagnosing renal infarction, as it directly visualizes the renal artery’s morphology, degree of stenosis, and presence of thrombosis, which is essential for determining vascular occlusion at the infarct site and guiding treatment planning. However, renal arteriography is an invasive procedure that carries certain risks, so it is typically not the first-choice diagnostic method and is reserved for cases in which other diagnostic techniques are inconclusive or when intervention is required. In this case, the patient underwent 2 non-contrast CT scans, neither of which detected a renal infarction. Therefore, for patients with similar presentations, if non-contrast CT does not show abnormalities, it is essential to perform contrast-enhanced CT to avoid misdiagnosis.

Therapeutic options for acute renal infarction, such as thrombolysis, anticoagulation, or surgery, can minimize renal function loss. Selective arterial thrombolysis is considered an effective treatment for unilateral renal infarction and may also be beneficial for bilateral infarction. Data suggest that thrombolytic reperfusion therapy is effective only if administered within 12 hours of renal ischemia, when the ischemic renal tissue is still viable.^[10]^ If renal necrosis occurs, nephrectomy should be performed. In most patients, heparin anticoagulation followed by warfarin therapy resulted in favorable outcomes.^[4]^ Huang et al^[15]^ argued that early aggressive anticoagulation is fundamental for treating this condition, and the efficacy and prognosis of anticoagulation alone are comparable to those of combined thrombolysis and anticoagulation. In cases of idiopathic renal infarction, the recurrence rate of thrombotic events is very low,^[16]^ leading to debate about the necessity of long-term anticoagulation therapy.^[17]^ Some studies suggest that for idiopathic renal infarction with a low recurrence rate, long-term anticoagulation may only increase the risk of bleeding.^[10]^ In this case, it was suspected that the abdominal pain from 7 days earlier was caused by renal infarction, which likely occurred earlier. Since the infarction had been prolonged, vascular intervention was unlikely to be beneficial. The patient was treated with low molecular weight heparin. After discharge, the patient continued receiving oral rivaroxaban as anticoagulation therapy. One month later, renal vascular ultrasound showed no significant localized stenosis or dilation in the proximal renal arteries, and blood flow was well-preserved.

## 4. Conclusions

This case highlights that acute renal infarction can occur in young individuals who have previously appeared healthy. In such cases, patients with renal infarction may present with gastrointestinal symptoms like nausea, vomiting, and diarrhea, potentially leading to misdiagnosis as acute gastroenteritis. Elevated LDH levels are highly sensitive for detection of renal infarctions. Contrast-enhanced CT is more reliable for the diagnosis of renal infarction. For patients with persistent abdominal pain, nausea, vomiting, and diarrhea, it is essential to assess lactate LDH levels. If ultrasound or non-contrast CT does not show any abnormalities, contrast-enhanced CT should be performed promptly to avoid misdiagnosis. Early aggressive anticoagulation therapy is fundamental for the treatment of idiopathic renal infarction.

## Author contributions

**Conceptualization:** Yi Hu.

**Formal analysis:** Libin Liu, Jingjiao Chen, Na Shi.

**Writing – original draft:** Libin Liu.

**Writing – review & editing:** Xiaomeng Wang, Yi Hu.
